# Spiritually oriented therapy approach applying in complex rehabilitation of patients with mental disorders

**DOI:** 10.1192/j.eurpsy.2022.1918

**Published:** 2022-09-01

**Authors:** V. Mitikhin, A. Magay, L. Alieva, A. Nochevkina

**Affiliations:** FSBSI “Mental Health Research Centre”, Department Of Mental Health Services, Moscow, Russian Federation

**Keywords:** schizophrénia, rehabilitation, psychosocial, biopsychosociospiritual

## Abstract

**Introduction:**

Numerous studies point to a high effectiveness of psychosocial rehabilitation using psychopharmacological treatment, psychosocial methods and spiritually oriented care technologies.

**Objectives:**

Description of the experience of using a spiritually oriented approach in complex rehabilitation of patients with mental disorders.

**Methods:**

Clinical, pathopsychological, statistic. The study involved two groups of patients with illness (ICD-10 F 20.04 – F 20.05, F25.х, F21.3-21.4, F 33.4, F31.7, F32.2 – 18 patients) and comorbid addictive disorders (ICD-10 F10.2х1- F10.2х2 - 17 patients). “The Social Adjustment Scale–Self-Report SAS-SR” (М. Weissman, S. Bothwell, 1976); “Medical Outcomes Study 36-item short form health survey” (SF-36, John E.Ware, 1992); “Methodology of the severity of anti-drug potential” (Kopeyko G.I. et al, 2018); “The Scale of Religiosity” (Kaz’mina O.Y., 2000) were used to assess the effectiveness.

**Results:**

All patients received psychopharmacological treatment and participated in rehabilitation work in patient community organizations using psychosocial and spiritually oriented therapy. The rehabilitation program included psychoeducation, skills training, group and individual psychotherapy, social activity. Spiritually oriented assistance was realized in the tradition of dialogical approach (Florenskaya T.A., 1992) and included conversations on Evangelical topics, work in therapeutic groups on the principles of a religious community. Longer remission times, a social functioning improvement, a tendency to change lifestyle based on the values of religious worldview, anti-drug potential increase and a higher understanding of religious life with an orientation towards internal religiosity were revealed among the participants of complex rehabilitation program.

**Conclusions:**

Perspective of using biopsychosociospiritual approach in psychiatry in work with patients with schizophrenia and patients with comorbid addictive disorders was shown.

**Disclosure:**

No significant relationships.

